# Changes in Expression of Manganese Superoxide Dismutase, Copper and Zinc Superoxide Dismutase and Catalase in *Brachionus calyciflorus* during the Aging Process

**DOI:** 10.1371/journal.pone.0057186

**Published:** 2013-02-22

**Authors:** Jianghua Yang, Siming Dong, Qichen Jiang, Tengjiao Kuang, Wenting Huang, Jiaxin Yang

**Affiliations:** Jiangsu Province Key Laboratory for Biodiversity & Biotechnology and Jiangsu Province Key Laboratory for Aquatic Live Food, School of Biological Sciences, Nanjing Normal University, Nanjing, Jiangsu, People's Republic of China; CNRS, Université de Bourgogne, France

## Abstract

Rotifers are useful model organisms for aging research, owing to their small body size (0.1–1 mm), short lifespan (6–14 days) and the relative easy in which aging and senescence phenotypes can be measured. Recent studies have shown that antioxidants can extend the lifespan of rotifers. In this paper, we analyzed changes in the mRNA expression level of genes encoding the antioxidants manganese superoxide dismutase (MnSOD), copper and zinc SOD (CuZnSOD) and catalase (CAT) during rotifer aging to clarify the function of these enzymes in this process. We also investigated the effects of common life-prolonging methods [dietary restriction (DR) and resveratrol] on the mRNA expression level of these genes. The results showed that the mRNA expression level of MnSOD decreased with aging, whereas that of CuZnSOD increased. The mRNA expression of CAT did not change significantly. This suggests that the ability to eliminate reactive oxygen species (ROS) in the mitochondria reduces with aging, thus aggravating the damaging effect of ROS on the mitochondria. DR significantly increased the mRNA expression level of MnSOD, CuZnSOD and CAT, which might explain why DR is able to extend rotifer lifespan. Although resveratrol also increased the mRNA expression level of MnSOD, it had significant inhibitory effects on the mRNA expression of CuZnSOD and CAT. In short, mRNA expression levels of CAT, MnSOD and CuZnSOD are likely to reflect the ability of mitochondria to eliminate ROS and delay the aging process.

## Introduction

Reactive oxygen species (ROS), such as hydrogen peroxide (H_2_O_2_) and superoxide anion (O_2_^−^), are produced mainly as by-products of aerobic respiration and can damage many cell macromolecules, including lipids, proteins and nucleic acids [1]. This damage has been hypothesized to be the major contributor to the aging process [1]–[4]. However, organisms have well-developed defense systems to eliminate ROS [5]. For example, antioxidant enzymes, such as superoxide dismutase (SOD) and catalase (CAT), catalyze the decomposition of ROS [6], [7], thereby moderating oxidative stresses and resulting in longer lifespan [8]. Previous studies have shown that SOD expression enhancement can extend the lifespan of the nematode *Caenorhabditis elegans* [9] and the fruit fly *Drosophila melanogaster* [10]. Rotifers are important members of freshwater zooplankton communities and, because of their ecological significance, are increasingly being used in ecotoxicological studies [11], [12]. In addition, they are often used in aging research, because of their small body size (0.1–1 mm in length) and short lifespan (6–14 days). Studies have also shown that antioxidants can extend the rotifer lifespan [13], which suggests that genes encoding antioxidant enzymes have an important role in rotifer aging. However, there are few reports of changes during aging in the expression of genes encoding antioxidant enzymes. The rotifer *Brachionus calyciflorus* has a short lifespan (5–7 days) and it is easy to detect changes in gene expression during the aging process in this species. Therefore, we analyzed changes in the mRNA expression level of genes encoding the antioxidants manganese SOD (MnSOD) [14], copper and zinc SOD (CuZnSOD) and CAT (GenBank accession no. JX000005, JX515577 and JX515578, respectively) in *B. calyciflorus* individuals of different ages to explore the role of such enzymes in the aging process.

Dietary restriction (DR) is usually defined as a moderate (normally 20–40%) reduction of available nutrients without causing malnutrition. It was first noted during the 1930s that DR significantly extended the lifespan of rodents [15]. In the laboratory, DR is one of the most commonly used life-history alterations among evolutionarily distinct eukaryotes, from single-cell to multicellular organisms [16]–[19]. Previous studies have suggested that DR can extend the lifespan of rotifers and enhance their resistance to oxidative stress [20]–[24]. Interestingly, DR-induced longevity and other beneficial effects of DR can transmit to the next generation in rotifers [25]. Resveratrol (3,5,40-trihydroxy-trans-stilbene) is a polyphenolic phytoallexin found in a variety of plant products [26], and has been reported to have beneficial effects on lifespan in many organisms, including yeast *Saccharomyces cerevisiae* [26], *D. melanogaster*, *C. elegans* [27], [28] and the fish *Nothobranchius furzeri* [29]. It also has been reported that resveratrol can protect against ROS-induced cell death by inhibiting ROS formation [30], preventing mitochondrial dysfunction [31] and restoring SOD activity [32].

The objective of present study was to investigate the effects of DR and resveratrol on the expression levels of the genes encoding MnSOD, CuZnSOD and CAT. Lifespan extension is always accompanied by an increase in viability in wide variety of species. Therefore we exposed the rotifers pretreated with DR and resveratrol to oxidative stress, and analyzed their survival.

## Materials and Methods

### Rotifers

The animals used during the study, *Brachionus calyciflorus* (Pallas), were neonate females (0–2-h old) hatched from resting eggs in artificial freshwater medium (EPA medium, pH ∼7.8) that comprised 96 mg NaHCO_3_, 60 mg CaSO_4_·H_2_O, 123 mg MgSO_4_, and 4 mg KCl in 1 L deionized water at 25°C [33]. Members of this species were originally collected in Gainesville, Florida in 1983 [34] and, since then, have been cultured continuously in the laboratory with periodic collection and storage of resting eggs. The females were fed by suspending the green alga *Chlorella pyrenoidosa* (Institute of Hydrobiology, Chinese Academy of Sciences, Wuhan, China) in the culture media at ∼3×10^6^ cells per mL. The feeding alga was cultured in BBM (Bolds Basal Medium) medium at 25°C in 3 L flasks under constant fluorescent illumination at 2000 lux.

### Experimental design

#### Age-related changes in antioxidant enzyme gene expression

In total, 600 neonates (0–2-h old) were collected and divided into six groups of 100 individuals, which were then cultured in 50 mL of prepared media maintained at 25°C for 0.5, 1, 2, 3, 4 and 5 days. Each treatment had three replicates. Rotifers were transferred into new medium every 24 h, and checked every 12 h to record the number of neonates, and the neonates were removed each day. Any surviving rotifers were then collected after 0.5, 1, 2, 3, 4 and 5 days. The total RNA was then extracted and reverse-transcribed into cDNA. Real-time PCR was carried out to measure the expression levels of MnSOD, CuZnSOD and CAT at different points of time above. The real-time PCR method was as described below.

##### Culture in 5 µM resveratrol

In total, 500 rotifers (0–2 h old) were collected and divided into five groups of 100 individuals, which then cultured in 50 mL of resveratrol (5 µM) media. As above, each treatment had three replicates. Culture plates were maintained at 25°C in darkness. The same number of rotifers was cultured in normal media as a control. As above, rotifers were transferred into new resveratrol medium every 24 h, checked every 12 h to record the number of neonates, and the neonates were removed each day. Rotifers were collected at 12, 24, 48, 72 and 96 h and the total RNA was then extracted. Resveratrol was obtained from Sigma Chemical Company (St. Louis, MO, USA), and was dissolved in acetone at a concentration of 50 mM and stored at −20°C. Final concentrations of resveratrol in the test medium were 5 µM and the concentration of acetone was 0.25%. This concentration of acetone had no detectable physiological effect on the rotifers [35].

##### Dietary restriction

Rotifers hatched from resting eggs were cultured for 24 h in normal media. In total, 100 rotifers were then selected at random and transferred into 50 mL of DR medium (i.e. no food) and starved for 12, 24 and 48 h. As above, each treatment had three replicates. Rotifers were transferred into newly prepared culture media every 24 h and checked every 12 h to remove neonates. Rotifers were collected at 12, 24 and 48 h and the total RNA was then extracted.

##### Oxidative stress resistance in rotifers pretreated with DR or resveratrol

Rotifers hatched from resting eggs were pretreated by either DR (i.e. provided with food once every 12 h) or resveratrol (5 µM), and were then transferred into paraquat (15.25 mg/L) media. Ten rotifers were added to six-well culture plates that contained 5 mL of the test solution. Each treatment had three replicates. The number of rotifers surviving after 12 h and 24 h was recorded. Resveratrol was dissolved in acetone and stored at −20°C. Paraquat (DMSO stock) was obtained from the Jiangsu Academy of Agricultural Sciences (Nanjing, China).

##### Age-related changes in swimming speed

To measure the rotifer swimming speed, we modified the method of Snell et al. (2011) as follows [13]. Experiments were carried out in a 48-well plate (10.20 mm in diameter) that contained 100 µL EPA. Video footage of rotifers swimming at ×4 magnification was captured with a camera attached to a LEICA EZ4D stereomicroscope. AutoCAD 2011 software was used to estimate the swimming speed (mm/s) of individual rotifers. From each age groups (0, 1, 2, 3, 4 and 5 days), eight to ten randomly selected rotifers were each videoed swimming for at least 1 min to measure the swimming speed.

##### Quantitative analysis of gene transcription

Total RNAs were extracted using the procedures described above. Total RNA was reverse-transcribed into cDNA using the PrimeScript RT Reagent Kit (TaKaRa, Japan). Real-time PCR was performed on a Mastercycler Ep Realplex (Eppendorf, Germany) to study the gene expression. The PCR reaction was performed in a 25 µL volume with a SYBR Premix Ex Taq™Kit (TaKaRa, Japan), 2 µM of each specific primer, and 1 µL of cDNA using the following procedure: initial denaturation at 95°C for 2 min followed by 40 cycles of amplification (95°C for 10 s and 55°C for 30 s). Table 1 shows the primers used in the quantitative real-time PCR. The gene encoding β actin (GenBank accession no. JX441322) was used as the internal control. The relative expression levels of the different genes were calculated using the 2^−ΔΔCT^ method [36].

**Table 1:** Quantitative real-time PCR primers used in the experiment.

Primers	Sequence (5'–3')
SOD-RTF	TTGCTTGTTCAAAACATACTC
SOD-RTR	TAATTGCTTCAGCTAAGTCTCC
CuZn SOD -RTF	GCCTTGACTCTCCTGTAC
CuZn SOD -RTR	GCTCGTCCTATTATAGAATGTG
CAT-RTF	ACAAGGCAATCCAGTCTATT
CAT-RTR	AATCGTCCAACAGGTATCAA
β Actin-RTF	GAAATTGTGCGCGACATCAAGGA
β Actin-RTF	GCAATGCCCGGGTACATGGTGGT

### Statistical analysis

Data were collected that represented the mean ± standard error (S.E.). All the statistical analyses were carried out with the SPSS 16.0 analytic package. Statistical significance was determined by one-way analysis of variance (ANOVA) and post-hoc Duncan multiple range tests. Significance was set at *P*<0.05. All the figures were produced using Sigma Plot version 11.0.

## Results

### Aging-related changes in rotifer phenotype

**Figure 1:**
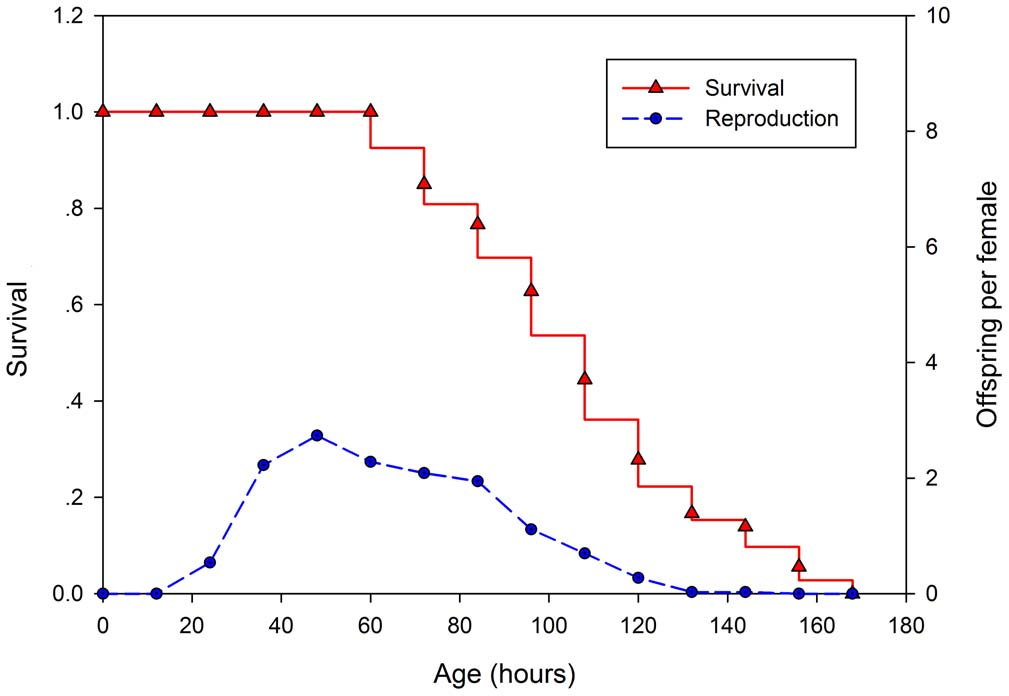
Typical survival and fecundity curves for *B. calyciflorus* under control conditions.

An example of a *B. calyciflorus* survivorship curve and fecundity schedule under control conditions is shown in Figure 1. Under these experimental conditions (25°C, *ad libitum* food), animals began reproducing after a short juvenile period of 18–20 h. Each rotifer was able to produce at least one generation and to carry no more than two unfertilized eggs 24 h post-hatching. Reproduction peaked at the age of 2 days, and was recorded as being approximately three offspring per female per day. Each female produced an average of 15.5 offspring in her lifetime. Under these conditions, rotifer reproduction was exclusively parthenogenetic. The first death occurred at 72 h, and numbers gradually increased with increasing age of the remaining rotifers. Under normal experimental conditions, the mean rotifer lifespan was 102 h.

**Figure 2:**
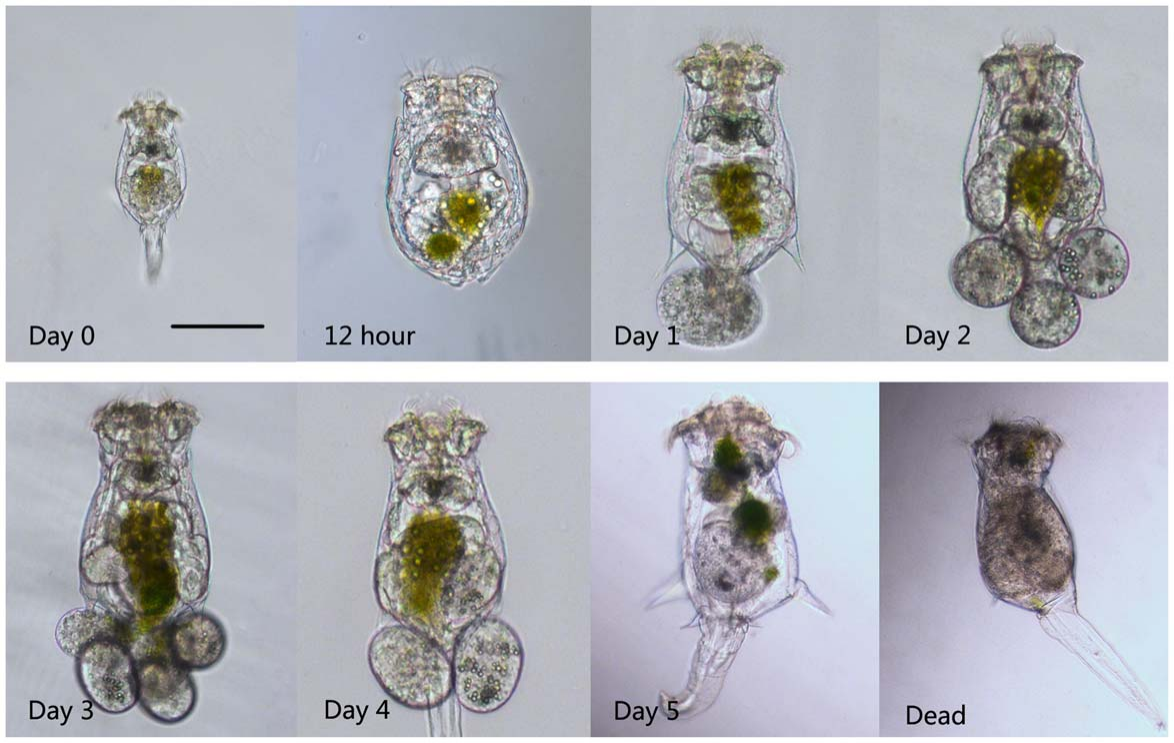
Photomicrographs of female rotifers of various ages.

**Figure 3:**
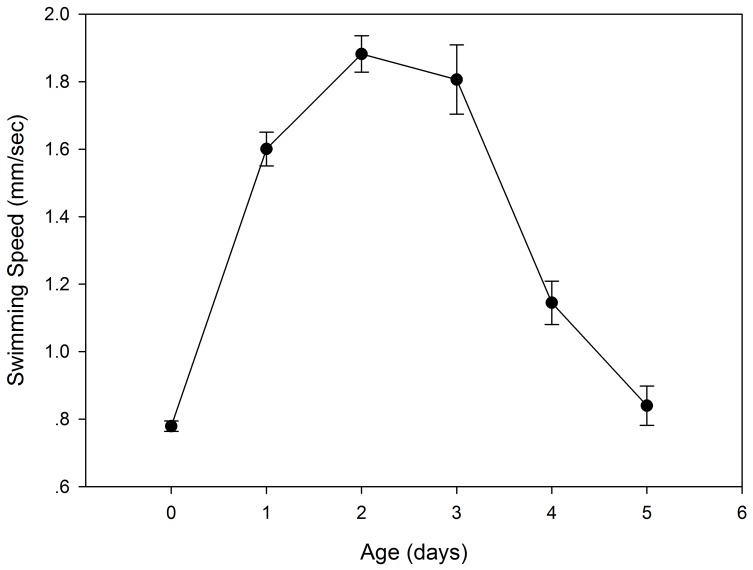
*B. calyciflorus* swimming speed (mm/s).

Figure 2 illustrates the aging phenotypes over the *B. calyciflorus* lifespan. Newly hatched rotifers were ∼180 µm long and grew to ∼300 µm in length after ∼12 h, when they matured into adults. This increase in size is known to be the result of water uptake and cytoplasm synthesis without cell division, given that rotifers are eutelic [13]. The study rotifers usually carried 1 egg at 1-day-old, and three or more (4–6) eggs at 2–3-days old. After the fourth day, their reproduction began to decrease. Egg production is a prominent feature of females because eggs are carried at the posterior of the females until they hatch [13]. On the fifth day, the rotifers generally did not reproduce, and their morphology changed significantly; for example, the spines on the posterior lengthened in some individuals. Swimming speed also changed significantly with the aging process (Figure 3). Newly hatched rotifers swam at 4.3-body lengths/s (∼0.78 mm/s). On the second day, the swimming speed peaked (1.89 mm/s, ∼6.3-body lengths/s) and then began to slowly decline.

### Age-related changes in antioxidant enzyme gene mRNA expression

**Figure 4:**
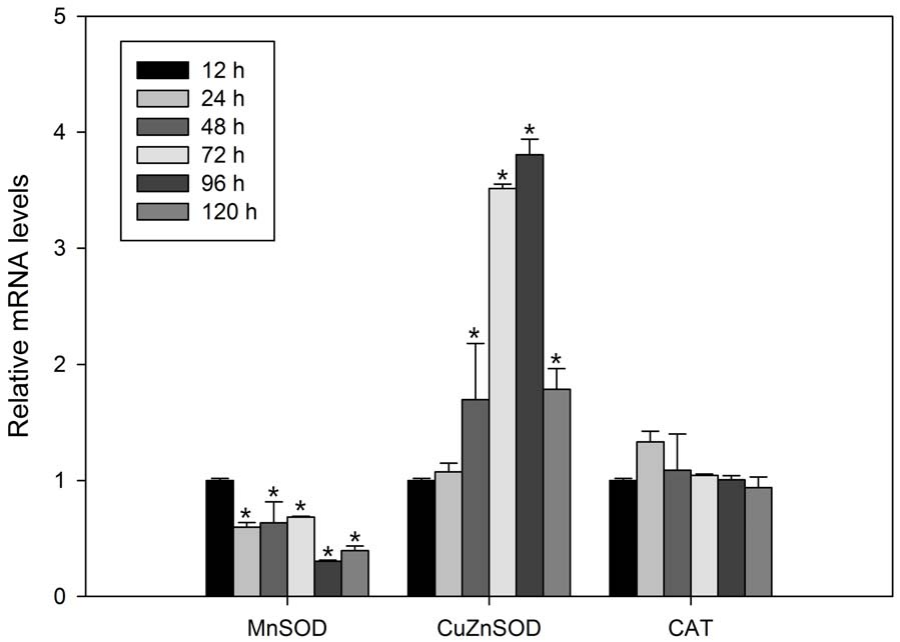
Relative mRNA expression levels of MnSOD, CuZnSOD and CAT from the *B. calyciflorus* at various ages. Each group contained 100 individuals. Bars represent standard errors. The expression levels were statistically analyzed by one-way ANOVA with the Tukey test. (^*^ *P*<0.05).

The changes in antioxidant enzyme gene expression are illustrated in Figure 4. The mRNA expression level of MnSOD decreased with aging (*P*<0.05), whereas that of CuZnSOD increased significantly (*P*<0.05). The expression of CAT did not change significantly (*P*>0.05).

### Egg-related changes in antioxidant enzyme gene mRNA expression

**Figure 5:**
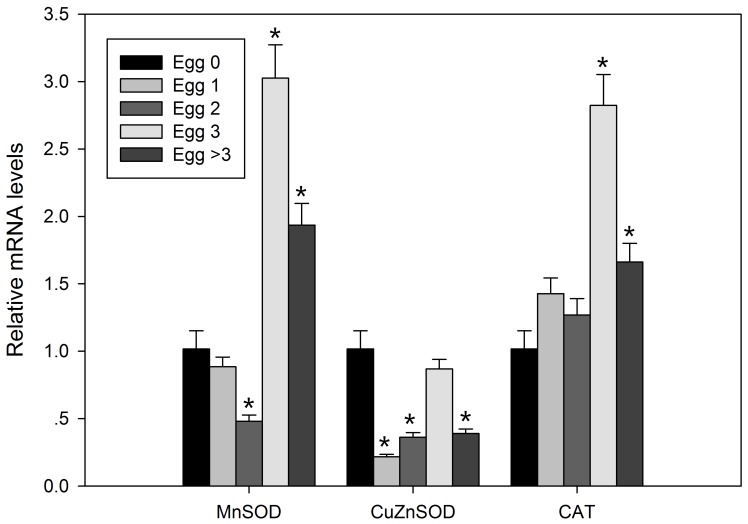
Relative mRNA expression levels of MnSOD, CuZnSOD and CAT from female *B. calyciflorus* carrying different numbers of eggs. Bars represent standard errors. Egg 0, Egg 1, Egg 2, Egg 3 and Egg >3 represent rotifers carry 0, 1, 2, 3 and 4 or more eggs, respectively. The expression levels were statistically analyzed by one-way ANOVA with the Tukey test. (^*^ *P*<0.05).

In rotifers carrying at least three eggs, MnSOD and CAT mRNA expression levels were significantly higher (*P*<0.05); by contrast, the mRNA expression of CuZnSOD decreased (*P*<0.05; Figure 5).

### The effects of resveratrol and DR on antioxidant enzyme gene mRNA expression

**Figure 6:**
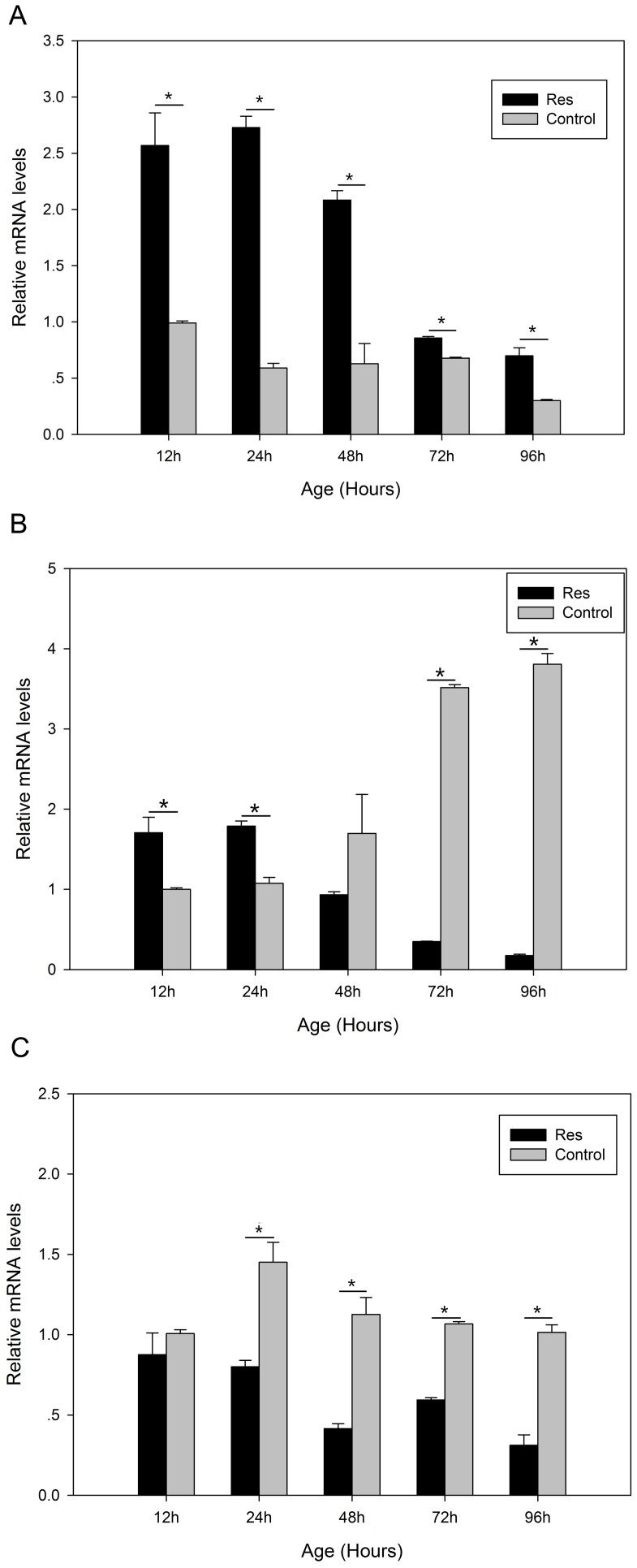
Relative mRNA expression levels of genes encoding the antioxidant enzymes. **(a)** manganese superoxide dismutase (MnSOD), **(b)** copper and zinc SOD (CuZnSOD) and **(c)** catalase (CAT) from *B. calyciflorus* rotifers treated with resveratrol. The concentration of resveratrol was 5 µM/L. Rotifers cultured in normal medium were used as the control. Each group contained 100 individuals. Bars represent standard errors. The expression levels were statistically analyzed by one-way ANOVA with the Tukey test. (^*^ *P*<0.05).

**Figure 7:**
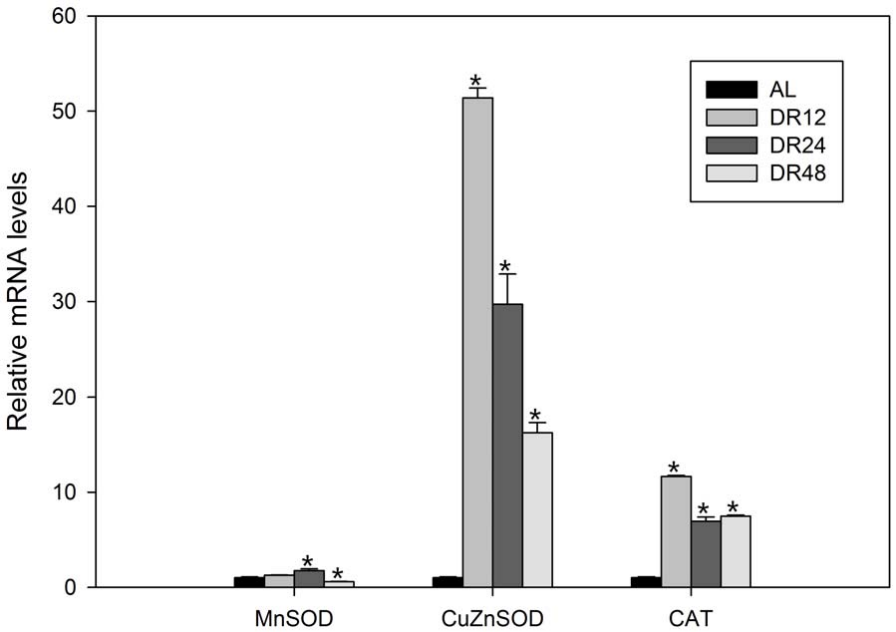
Relative mRNA expression levels of MnSOD, CuZnSOD and CAT from *B. calyciflorus* under various DR conditions. AL, DR12, DR24 and DR48 represent rotifer groups starved for 0, 12, 24 and 48 h, respectively. Bars represent standard errors. The expression levels were statistically analyzed by one-way ANOVA with the Tukey test. (^*^ *P*<0.05).

Quantitative real-time PCR showed that resveratrol significantly increased (*P*<0.05) the mRNA expression of the gene encoding MnSOD (Figure 6a), whereas the expression levels of the genes encoding CuZnSOD (Figure 6b) and CAT (Figure 6c) were inhibited. The mRNA expression of MnSOD was enhanced after 24-h DR, but was significantly lower (*P*<0.05) after 48-h DR (Figure 7). Starvation also increased the mRNA expression of the genes encoding CuZnSOD and CAT, which peaked after 12-h DR (Figure 7).

### Effect of DR and resveratrol on rotifer swimming speed at 4-days-old

**Figure 8:**
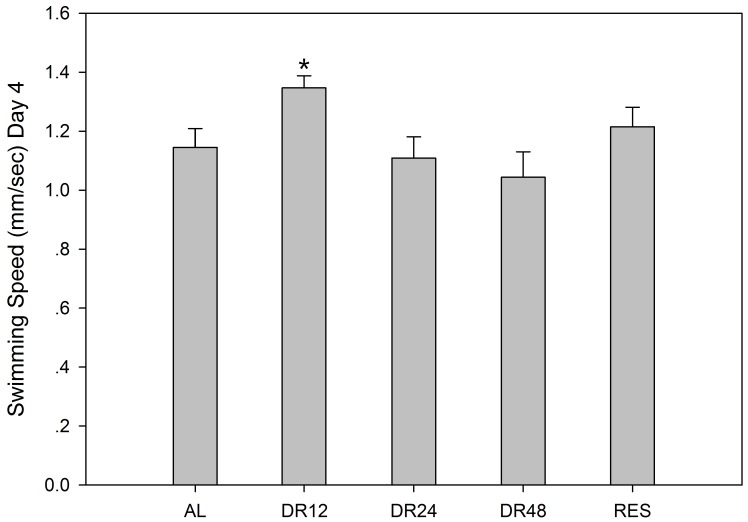
The swimming speed of 4-days-old *B. calyciflorus*. AL, DR12, DR24 and DR48 represent the rotifer groups starved for 0 h, 12 h, 24 h and 48 h, respectively. Bars represent standard errors. The asterisk indicates significant difference from control. (^*^ *P*<0.05).

Resveratrol treatment did not increase rotifer swimming speed of 4-days-old rotifers, whereas moderate DR (12 h) increased the swimming speed (*P*<0.05). In the groups starved for 24 h and 48 h, there was no significant change in swimming speed (*P*>0.05; Figure 8).

### Effect of DR and resveratrol pretreatment on oxidative stress resistance

**Figure 9:**
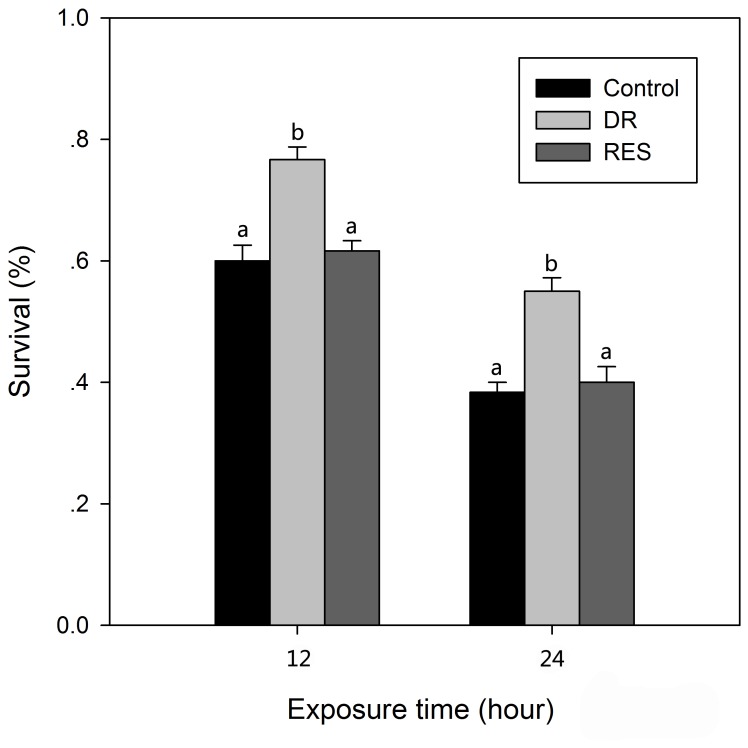
The survival rate of *B. calyciflorus* under oxidative stress conditions (paraquat). The survival rate was statistically analyzed by one-way ANOVA and post-hoc Duncan multiple range test. The different letters indicate a significant difference for each time point with control (*P*<0.05).

Figure 9 illustrates the effects of DR and resveratrol pretreatments on the survival rate of rotifers under oxidative stress conditions. After being exposed to oxidative stress for 12 h, the survival rate of the DR pretreated group was 0.78±0.02, significantly higher (*P*<0.05) than of the control group (0.60±0.25). A similarly significant result was recorded for the group exposed for 24 h: the survival rates of the DR pretreated and control groups were 0.55±0.02 and 0.38±0.02, respectively (*P*<0.05). This result indicates that DR pretreatment can increase the rotifer survival rate under paraquat-induced oxidative stress. However, resveratrol pretreatment did not significantly improve survival under oxidative stress.

## Discussion

The accumulation of ROS, which are the main by-products of aerobic respiration, impairs the function of cellular macromolecules, including lipids, proteins and DNA, and is considered to be one of the main causes of aging [1]. Animals have evolved defenses against ROS damage, and the balance between oxidative stress and concentration of available antioxidants has a major influence on lifespan [13]. Previous studies have demonstrated that high expression of SODs could extend the lifespan of several organisms [10], [37]. Although the mechanism of antioxidant effects on animal aging remains controversial [38], the ability of antioxidants to extend rotifer lifespan has been confirmed [13]. In this study, we investigated how antioxidant enzyme gene mRNA expression changes with age, and our results are likely to be useful for further studies on the role of antioxidant genes in aging. The mRNA expression levels of MnSOD decreased with aging, whereas those of CuZnSOD increased. However, the mRNA expression level of CAT did not change significantly. MnSOD (SOD, E.C. 1.15.1.1) is a mitochondrial protein that catalyzes the conversion of O_2_^−^ to the less reactive H_2_O_2_ [6]. MnSOD is considered to be the most important isoform for the regulation of lifespan, because mitochondria are the main source of ROS [24]. Therefore, decreases in MnSOD expression are not conducive to the elimination of ROS. CAT (E.C. 1.11.1.6), another antioxidant enzyme containing heme, catalyzes the degradation of H_2_O_2_ to water (H_2_O) and molecular oxygen (O_2_) [24], [39]. CAT is also an enzyme with one of the highest turnovers and is important in reproductive reactions. CuZnSOD, which is localized in cytoplasm, contains copper and zinc at the catalytic centers and also catalyzes O_2_^−^ to H_2_O_2_ [6]. Given that ROS are mainly produced inside organelles, such as mitochondria [40], [41], such cellular bodies are among the first to be damaged by ROS. In addition, accumulating oxidative damage can affect the efficiency of mitochondria and further increase the rate of ROS production [42]. Thus, increased expression of MnSOD and CAT retards the aging process and extends lifespan, as shown by several overexpression studies in *D. melanogaster* [43], [44] and mouse *Mus musculus* [45]. The reduction of MnSOD expression level with age will aggravate ROS-induced mitochondrial damage. Although CuZnSOD expression increased with age, it can only have an effect in the cytoplasm. Snell and co-workers measured the concentration of ROS in rotifers of different ages and found no increase in ROS load with aging [13]. Therefore, ROS damage accumulation might be a major contributor to the aging process [1]–[4], [46].

It is a widespread phenomenon that many organisms, including *C. elegans* [47], *D. melanogaster* [48] and rodents [49], [50] would increase their lifespan and/or reduce their reproductive rate when food is less available [51]. Therefore, there is likely to be a close relationship between aging and reproductive effort. Lifespan elongation and fecundity reduction was also observed when rotifers were denied food [22], [51], [52], which suggests that suspension or slowdown of reproduction can retard the rate of aging process and prolong lifespan. The present study shows that in rotifers carrying three or more eggs, MnSOD and CAT expression levels were significantly higher (Figure 5). This result suggests that reproduction increases oxidative stress and influences the expression of antioxidant enzyme genes. This would be a reason that accelerates the aging process of rotifers. However, the relation between reproduction and aging is intricate and requires further study as to whether aging is mainly or directly caused by reproduction in rotifers.

DR is a well-known method of increasing both lifespan and resistance to exogenous oxidative stress provoked by ROS generators [53]. In rotifers, DR influences the expression of many genes, including those encoding SOD [8], [25], CAT [25], heat shock protein (HSP)-70 and Glucose-regulated protein (GRP)-94 [54], and extends lifespan [20]–[22], [55]. In our study, we found that moderate DR (12 and 24 h) increased the expression of the genes encoding MnSOD, CuZnSOD and CAT (Figure 7). This antioxidant gene expression enhancement could, to some extent, explain why DR extends lifespan. However, aging is likely to be regulated by multiple, low-affinity, low-specificity interaction processes, mediated through a complex tangle of overlapping pathways [13]. Antioxidant genes might be just one part of the whole system.

Many of the polyphenols such as resveratrol and quercetin, are synthesized by plants under different stresses (e.g. infection, starvation or dehydration) [53], [56]. Resveratrol has beneficial effects on lifespan in a range of organisms, from yeast to mammals [26]–[29], [57], [58], through a DR-like mechanism. This is dependent on an NAD^+^-dependent histone deacetylase, Sir2 in *Drosophila* and SIR-2.1 in *C. elegans* [59]. Resveratrol acts as a strong radical scavenger and regulates SOD expression in nematodes [60] and rats [32]. In our study, resveratrol did not extend the lifespan of the rotifers (data not shown), although it did induce MnSOD expression, the high levels of which might contribute to reducing the damage caused by ROS in mitochondria [61]. However, resveratrol might inhibit CuZnSOD and CAT expression, thus resulting in aggravating ROS damage to other organelles. This might explain why resveratrol cannot extend the lifespan of rotifers. Moreover, resveratrol did not improve the swimming speed of rotifers (at 4-days-old), which further confirms the fact that resveratrol has no effect on the rotifer aging process (Figure 8). Snell and co-workers found that dietary exposure to only certain antioxidants extended rotifer lifespan [13]. This phenomenon suggests that the mechanism of antioxidant action is still unclear.

To some extent, high levels of antioxidant enzyme gene expression might convey greater resistance to oxidative stress. To assess the resistance to oxidative stress in rotifers, we used paraquat, a widely used compound that generates ROS inside the cell [25]. Under this oxidative stress, DR pretreatment increased the rotifer survival rate. However, resveratrol pretreatment had no effect on rotifer survival rate under oxidative stress. DR treatment increased the expression of genes encoding all three antioxidant enzymes (MnSOD, CuZnSOD and CAT), whereas resveratrol treatment only increased expression of the gene encoding MnSOD. We believe that inducing the expression of genes encoding antioxidant enzymes would contribute to improving the ability of an organism to resist oxidative stress.

In this study, we examined the effects of aging on the expression of genes encoding antioxidant enzymes in rotifers. Our results suggest that the decrease in antioxidant gene expression is one of the causes of aging. Lifespan extension by DR might also be associated with an increase in oxidation resistance. Resveratrol, as an exogenous antioxidant, can facilitate the expression of MnSOD only, although its inhibition of CuZnSOD and CAT might be one of the reasons why lifespan did not extend in those rotifers treated with this compound.
